# Immune Checkpoint and Telomerase Crosstalk Is Mediated by miRNA-138 in Bladder Cancer

**DOI:** 10.3389/fonc.2021.795242

**Published:** 2022-02-11

**Authors:** Hajar El Ahanidi, Meryem El Azzouzi, Chaimae Hafidi Alaoui, Mohammed Tetou, Mounia Bensaid, Imane Chaoui, Laila Benbacer, Ilias Hassan, Mohamed Oukabli, Katarzyna Michaud, Ahmed Ameur, Abderrahmane Al Bouzidi, Mohammed El Mzibri, Camilla Jandus, Mohammed Attaleb

**Affiliations:** ^1^ Biology and Medical Research Unit, Centre National de l'Energie, des Sciences et Techniques Nucleaires (CNESTEN), Rabat, Morocco; ^2^ Rabat Medical and Pharmacy School, Mohammed V University in Rabat, Rabat, Morocco; ^3^ Department of Pathology and Immunology, University of Geneva, Geneva, Switzerland; ^4^ Faculty of Sciences, Mohammed V University in Rabat, Rabat, Morocco; ^5^ Military Hospital Mohammed V, Rabat, Morocco; ^6^ University Center of Legal Medicine Lausanne-Geneva, Lausanne University Hospital, University of Lausanne, Lausanne, Switzerland; ^7^ Ludwig Institute for Cancer Research, Lausanne Branch, Lausanne, Switzerland

**Keywords:** bladder cancer, TERT, immune checkpoint, microRNAs, immunotherapy

## Abstract

**Background:**

Tumor recurrence and progression in non-muscle invasive bladder cancer (NMIBC), therapy failure, and severe side effects in muscle invasive bladder cancer (MIBC) are the major challenges in the clinical management of bladder cancer (BC). Here, we identify new molecular targetable signatures to improve BC patients’ stratification and the outcome of current immunotherapies.

**Material and Methods:**

In a prospective cohort of 70 BC patients, we assessed the genetic and molecular regulation of TERT in maintaining telomere length in parallel to immune checkpoint and microRNA expression.

**Results:**

TERT was undetectable in healthy bladder tissues but upregulated in invasive BC stages and high tumor grade. Its expression was linked with the combined effect of the C250T mutation and THOR hypermethylation, associated with progressing tumors and maintaining of telomere length. In the same cohort, PD-L1 scored highest in NMIBC, while PD-L2 was upregulated in MIBC. We also show that miR-100-5p and 138-5p were highly expressed in healthy bladder specimens and cell line, while expression decreased in the BC tissues and BC cell lines. In line with the binding prediction for these miRNAs on target genes, miRs 100-5p and 138-5p expression strongly inverse correlated with TERT, PD-L1, and PD-L2 expression, but not PD1.

**Conclusion:**

We identify a loop involving TERT, PD1-ligands, and miR-138-5p in BC, that might represent not only a useful biomarker for improved diagnosis and patients’ stratification but also as a promising axis that might be therapeutically targeted *in situ*.

## Introduction

Among all the tumors of the urinary tract that fall under the purview of urologists, urothelial carcinoma (UC) is the one that is frighteningly common while still being deadly (1). Bladder cancer (BC) is the most frequent UC and ranks as the ninth most frequently diagnosed cancer worldwide (2). BC is a heterogeneous epithelial malignancy with more than 90% being urothelial and about 5% squamous cell carcinoma (3, 4). BC progresses along two distinct pathways that pose different challenges for clinical management. Non-muscle invasive bladder cancer (NMIBC) is relatively benign but tends to recur (5). The current first-line therapy for NMIBC (intermediate- and high-risk papillary tumors and carcinoma *in situ*) consists in intravesical instillations of Bacillus Calmette-Guerin (BCG) as it significantly reduces the risk of progression to muscle invasive disease as it significantly reduces the risk of progression to muscle invasive disease. However, the high recurrence rate necessitates radical cystectomy or enrollment in clinical trials (6–8).

While low-risk (i.e., solitary, G1 Ta) and high-risk (i.e., any T1, G3 or CIS) diseases have been well-defined using the TMN staging system and the 1973 and 2004 WHO grading classifications, the intermediate-risk category has traditionally comprised all patients not included in either of these categories (9).

Muscle invasive bladder cancer (MIBC) has a propensity to metastasize and is treated by chemotherapy, cystectomy, and more recently using immune checkpoint inhibitors (ICIs) (7).

Immune checkpoint inhibitors are approved by the US Food and Drug Administration for MIBC patients who progress despite platinum-based chemotherapy or as first-line treatment for cisplatin-ineligible patients (10–12). Yet, overall response rates do not exceed 25% (13). In that regard, it has been shown that high PD-L1 expression is associated with higher stage and grade, higher recurrence, poor survival, and worse outcome in BC (14, 15). However, low PD-L1 expressing tumors can respond to checkpoint inhibition, while inversely, some patients with high PD-L1 levels do not, underscoring the need to better understand the mechanisms involved in the response to these agents (16), including tumor mutational burden and epigenetic dysregulations (11).

The search for non-invasive methods in the diagnosis and monitoring of BC highlighted the potential of urines as valuable matrix for detecting molecular changes in the bladder (17). Liquid biopsy markers such as exfoliated bladder cancer cells, cell-free DNA (cfDNA), proteins, and exosomes have the potential to translate the changes in the bladder and inform about the nature of the disease, its management and care, as they allow a cost-effective and convenient mode of patient monitoring (18). While cfDNA provides an accurate risk stratification tool and might be essential for determining the best surveillance strategy (19), urine micro-RNAs (miRNAs) have the potential to be a valid marker for BC detection and can successfully compete with other non-invasive diagnostic tests (20).

In the context of miRNAs, a large study using the cancer genome atlas data predicted a list of 1,381 miRNAs, as potential epigenetic regulators for multiple pathways (21). These miRNAs were related to mitogen-activated protein kinase (MAPK) signaling, apoptosis, and tumor necrosis factor (TNF) pathways, all regulators of cancer development, progression, and immune escape. More stringent studies showed that low miR-100 expression correlates with longer progression-free survival (PFS) and cancer-specific overall survival in BC (22). In colorectal cancer, miR-138 downregulation was observed in parallel to telomerase reverse transcriptase (TERT) upregulation, suggesting that miR-138-5p works at least partially through TERT downregulation, thus acting as tumor suppressor with the potential to inhibit telomerase expression (23, 24).

Telomerase activity is found in ~90% of human carcinomas and, of particular interest, ~99% of UC exhibit a high telomerase activity (25), conferring to tumor cells replicative immortality (26). Telomerase is responsible for elongating telomeres through re-expression of the catalytic subunit of TERT (27). It is active in stem cells, while in developing tissues, levels of telomerase activity decrease (28). The mechanisms of TERT induction in cancer cells are numerous and diverse, including promoter mutation (29) and epigenetic regulation (26). While hypermethylation induces downregulation in most genes, the hypermethylation of TERT favors expression. Indeed, a unique feature of TERT promoter arises on its unmethylation state in normal cells and its methylation in malignant cells (30). In that regard, in colorectal cancer, it was shown that THOR (TERT Hypermethylated Oncological Region) hypermethylation is closely associated with the increase of TERT expression (31) and that demethylation of the telomerase catalytic site decreased the telomerase activity and remarkably shortened telomeres (32).

A retrospective study in advanced UC investigating the impact of mutational burden on patients treated with ICIs showed that the presence of a TERT promoter mutation was an independent predictor of improved PFS and overall survival, rising questions about the involvement of other regulator of IC and calling for additional genomic profiling in the clinical decision making (13).

Given the lack of comprehensive studies exploring the TERT behavior and the expression of IC in the same set of BC tumors, we performed a prospective study investigating the hotspot mutations and the methylation status of TERT promoter, gene expression level, and their relationship with telomere length in parallel to the expression of PD1, PD-L1, and PD-L2 in the same cohort of 70 BC patients. Moreover, we provide a novel potential link between the miRNAs 100-5p and 138-5p and the TERT/PD-L1/PD-L2 loop.

## Material and Methods

### Tissue Sampling and Patients’ Characteristics

A total of 70 fresh frozen tumor samples were obtained by transurethral resection of the bladder or cystoscopy at the Urology Department of University Military Hospital in Rabat, Morocco. Patient eligibility criteria included histologically confirmed BC and follow-up data availability. Patients presenting with other cancer types at the time of enrollment were excluded.

The study protocol (N82/19) was approved by the Ethics Committee for Biomedical Research from the Faculty of Medicine and Pharmacy of Rabat-Morocco. All recruited patients who agreed to participate in the study, have been informed on the study and its objectives, and signed written consent. With respect to ethical rules, rights of confidentiality and anonymity of participants have been respected. The characteristics of the study population are reported in **Table 1**.

**Table 1:** The clinicopathological characteristics of BC patients included in this study.

Parameter	Total cases	Percentage %
**Sex**		
Male	68	97.14
Female	2	2.86
**Age**		
<50	1	1.43
50–70	44	62.86
>70	25	35.71
**Smoking history**		
Yes	28	40
No	42	60
**Tumor stage**		
≤PT1	52	74.29
>PT1	18	25.71
**Tumor grade**		
Low grade	27	38.57
High grade	43	61.43
**Tumor recurrence**		
Yes	12	23.08
No	40	76.92
**Tumor progression**		
Yes	5	9.62
No	47	90.38

Staging and grading were done at the Anatomopathology Department of the Military Hospital of Rabat, according to the TNM classification (NMIBC includes Ta, T1, and Tis; and MIBC includes T2, T3, and T4) and World Health Organization criteria. All patients were followed up in accordance with standard recommendations. Recurrence was defined as reappearance of primary NMIBC with a lower or the same pathological stage, and progression was defined as a disease with a higher TNM stage upon relapse (33).

Four healthy bladder specimens were taken from freshly deceased, healthy cadaveric donors at the University Center of Legal Medicine Lausanne-Geneva, Lausanne University, Hospital, University of Lausanne, Lausanne, Switzerland (VD431/13) and used as controls.

### Cell Culture

Five human bladder cell lines mimicking the healthy bladder state (HCV29), a non-muscle invasive bladder cancer stage (BU68.08, kindly provided by Dr. Laurent Derré, University Hospital Lausanne, Switzerland) and the three stages of invasiveness of the disease (RT4:pT2, J82:pT3, TCCSUP:pT4) were grown in RPMI 10% heat-inactivated FCS. Cells were grown at 37°C in a 5% CO2 humidified atmosphere and were used for experiments when confluency reached 70% to 80%. One million cells from each line were used for total RNA extraction or flow cytometry analysis.

### DNA and RNA Extraction

Genomic DNA was extracted from fresh frozen biopsies using phenol/chloroform method. Total RNA was extracted from fresh frozen biopsies conserved in RNA Later (Invitrogen, Waltham, MA, USA) and from five bladder cell lines using TRI Reagent (Sigma-Aldrich, Inc., St. Louis, MO, USA), according to the manufacturer’s instructions. Amount and quality of DNA and RNA were measured by NanoDrop 2000 (Thermo Fisher Scientific, Waltham, MA, USA).

The ratio of absorbance at 260 and 280 nm was used to assess DNA purity. A ratio of ∼1.8 was accepted as “pure” for DNA. RNA was considered DNA and protein free if the ratio of readings at 260/280 nm was ≥1.7.

### Evaluation of TERT Mutational Status

A 163-bp TERT promoter region was amplified using the primers shown in **Table 2**. PCR products were purified using the ExS-Pure™ enzymatic PCR cleanup kit (Nimagen, Nijmegen, The Netherlands) and sequenced on an ABI 3130XL DNA analyzer, using BigDye Terminator v3.1 Cycle Sequencing Kit (Applied Biosystems, Foster City, CA, USA). The obtained sequences were matched with the gene reference sequence (Gene ID: 7015) collected from the GeneBank database. The sequence alignment was performed using Clustal W program in BioEdit Software.

**Table 2:** Primers and annealing temperatures.

Analysis	Forward primer	Reverse primer	Tm (°C)
	5′–3′	5′–3′	
Mutational status	CACCCGTCCTGCCCCTTCACCTT	GGCTTCCCACGTGCGCAGCAGGA	68
MSP			
Methylated	GAGGTATTTCGGGAGGTTTCGC	ACTCCGAACACCACGAATACCG	58
Unmethylated	GGGAGGTATTTTGGGAGGTTTTGT	CAAACTCCAAACACCACAAATACCA	58
Telomere length	ACACTAAGGTTTGGGTTTGGGTTTGGGTTTGGGTTAGTGT	TGTTAGGTATCCCTATCCCTATCCCTATCCCTATCCCTAACA	60
TERT expression	CCGCCTGAGCTGTACTTTGT	CAGGTGAGCCACGAACTGT	60
Albumin	CGGCGGCGGGCGGCGCGGGCTGGGCGGAAATGCTGCACAGAATCCTTG	GCCCGGCCCGCCGCGCCCGTCCCGCCGGAAAAGCATGGTCGCCTGTT	60
PD1 expression	GCGGCCAGGATGGTTCTTAG	CCTTCGGTCACCACGAGCA	60
PD-L1 expression	GGACAAGCAGTGACCATCAAG	CCCAGAATTACCAAGTGAGTCCT	60
PD-L2 expression	ACCGTGAAAGAGCCACTTTG	GCGACCCCATAGATGATTATGC	60
B2M	GAGGCTATCCAGCGTACTCCA	CGGCAGGCATACTCATCTTTT	60

### Epigenetic Profiling

#### Sodium Bisulfite Modification

Genomic DNA extracted from tumors was subjected to bisulfite treatment to convert all unmethylated cytosine to uracil and keep the methylated cytosines unchanged. Overall, 500 ng of genomic DNA from each sample was used for bisulfite modification as previously described (34). Sodium bisulfite modification and DNA purification were performed using the EZ DNA Methylation Kit (Zymo Research, Orange, CA, USA).

#### Methylation-Specific PCR

The modified DNA was subjected to methylation-specific PCR (MSP) using unmethylation-specific or methylation-specific primers. The amplification reaction was carried out in a final volume of 25 µl containing 2 µl of modified DNA, 1.5 mM MgCl_2_, 10 µM of each dNTP, 10 µM of each primer, and 1 U of Platinum Taq Polymerase in 1× PCR buffer (Invitrogen, USA). PCR reactions were performed in thermal cycler (Gene Amp, PCR system 9700, Applied Biosystems, Cheshire, UK). Mixtures were first denatured at 95°C for 10 min. A total of 40 cycles of PCR were then performed with denaturation at 95°C for 30 s, primer annealing for 45 s at the corresponding temperature (**Table 2**), and primer extension for 45 s at 72°C. At the end of the last cycle, mixtures were incubated at 72°C for 10 min. For every reaction, a negative control, in which DNA template was omitted from the amplification mixture, was included. PCR products were analyzed by electrophoresis on 2% agarose gel followed by staining with ethidium bromide (10 mg/ml).

### Gene Expression Study

Total RNA was subjected to reverse transcriptase reaction using the high-capacity cDNA reverse transcription kit (Applied Biosystems, Cheshire, UK). cDNAs were subsequently used to perform a SYBR green-based quantitative real-time PCR using the KAPA SYBR FAST Kit (Roche, Basel, Switzerland). Samples were amplified simultaneously in triplicate in one-assay run with a nontemplate control for each primer pair to control for contamination or for primer dimerization. TERT, PD1, PD-L1, and PD-L2 levels were normalized to the expression of β2 microglobulin (β2M) used as internal control gene, using the 2^−ΔCt^ formula. Primer sequences are shown in **Table 2**.

### Telomere Length Measurement

Relative telomere length was assessed by q-PCR. Briefly, the telomere repeat copy number to single gene copy number (T/S) ratio was determined using an Applied Biosystems thermocycler. Briefly, 25 ng of genomic DNA resuspended in 10 μl of either the telomere or albumin PCR reaction mixture proceeded for 1 cycle at 95°C for 5 min, followed by 40 cycles at 95°C for 15 s, and 60°C for 1 min. All samples for both the telomere and single-copy gene (albumin) reactions were done in triplicate. In addition to the samples, each plate contained a 5-point standard curve from 5 to 100 ng using a pool of genomic DNA. The linear correlation coefficient (*R*2) value for both reactions ranged between 0.97 and 0.99. The primers used for the experiment are shown in **Table 2**.

### miRNA Quantification

The cDNA for the miRNA study was synthetized using TaqMan Advanced miRNA cDNA Synthesis Kit, and the quantification was performed with a real-time PCR using the standard protocol of TaqMan Fast Advanced Master Mix. TaqMan Advanced miRNA Assay has-100-5p and has-138-5p were used to quantify the target, and the has-16-5p was used as endogenous control.

### Flow Cytometry Analysis

Cells from the five bladder cell lines were washed and labeled during 20 min at RT with the following antibodies: Brilliant Violet 421 antihuman PD1 (329920, Biolegend, 1/100, San Diego, CA, USA), phycoerythrin antihuman PD-L1 (329706, Biolegend, 2/100), APC antihuman PD-L2 (329608, Biolegend, 2/100), or left unstained.

Dead cells were excluded using the viability dye DRAQ7 (Invitrogen). Samples were acquired on CytoFLEX flow cytometer (Beckman Coulter, Brea, CA, USA), and the analysis was performed on FlowJo software, version 10.7.1.

### miRNA Binding Assessment

MiRWALK 2.0 and miRTarBase 2020 databases and miRabel tool that compromises results from three different databases (miRanda, PITA, SVmicrO, and TargetScan) were used to predict the binding of the miR-100-5p and miR-138-5p on the target genes.

### Statistical Tests

Statistical analysis was performed using GraphPad Prism software version 8 using nonparametric tests (Mann-Whitney *U* or Kruskal-Wallis tests) and Pearson’s correlation test. A *p*-value <0.05 (two-tailed) was considered statistically significant and labeled with an asterisk. *p*-values <0.01, 0.001, or 0.0001 were labeled with ^**^, ^***^, or ^****^, respectively.

## Results

### TERT Is Upregulated in BC at Late Stages of Carcinogenesis

First, we performed sequencing of the TERT promoter in our cohort of 70 BC patients. Sequencing revealed that 60% of the bladder specimens had a mutation of the promoter region (42/70), with C228T being the most prevalent mutation found in 80.95% of cases (34/42). The other mutations, C250T, A161C, and G149T, were reported in 16.67% (7/42), 2.38% (1/42), and 2.38% (1/42) of cases, respectively. One case carried more than one mutation (C228T and C250T). Distribution of TERT promoter mutations according to clinicopathological features is reported in **Table 3** and showed that mutations in the promoter region of TERT gene are more prevalent in high-grade tumors (65.11%; 2/43) and in advanced stages (66.67%; 12/18).

**Table 3:** Distribution of mutations in the TERT gene promoter according to clinicopathological features.

Parameter	Overall		C228T		C250T		A161C		G149T	
	+	−	+	−	+	−	+	−	+	−
**Gender**										
Male	42	26	33	35	7	61	1	67	1	67
Female	1	1	1	1	0	2	0	2	0	2
*p*-value	0.751									
**Age**										
<50	1	0	1	0	0	1	0	1	0	1
50–70	28	16	24	20	3	41	0	44	1	43
>70	14	11	9	16	4	21	1	24	0	25
*p*-value	0.154									
**Smoking**										
Yes	18	10	16	12	1	27	0	28	1	27
No	25	17	18	24	6	36	1	41	0	42
*p*-value	0.941									
**Tumor stage**										
≤PT1	31	21	23	29	6	46	1	51	1	51
>PT1	12	6	11	7	1	17	0	18	0	18
*p*-value	0.860									
**Tumor grade**										
LG	15	12	11	16	2	25	1	26	1	26
HG	28	15	23	20	5	38	0	43	0	43
*p*-value	0.538									
**Tumor recurrence**										
Yes	7	5	6	6	1	11	0	12	0	12
No	24	16	17	23	5	35	1	39	1	39
*p*-value	0.647									
**Tumor progression**										
Yes	1	4	1	4	0	5	0	5	0	5
No	30	17	22	25	6	41	1	46	1	46
*p*-value	0.083									

Second, we investigated the methylation profile of the THOR region of TERT promoter and found it to be hypermethylated in 90% of cases (63/70). Distribution of the methylation status according to clinicopathological parameters is reported in **Table 4** and showed no significant association between the methylation status and sex, age, smoking, and tumor stage and grade. Indeed, hypermethylation of TERT promoter was observed in both high- (90.7%; 39/43) and low-grade tumors (88.89%; 24/27) and in both non-muscle invasive (90.38%; 47/52) and muscle invasive stages (88.89%; 16/18) (**Table 4**).

**Table 4:** Association between the methylation status of TERT gene promoter and clinicopathological features.

Parameter	TERT hypermethylation	TERT hypomethylation
**Gender**		
Male	61	7
Female	2	0
*p*-value	0.999	
**Age**		
≤50	1	0
50–70	41	3
>70	21	4
*p*-value	0.696	
**Smoking**		
Yes	25	3
No	38	4
*p*-value	0.945	
**Tumor stage**		
≤PT1	47	5
>PT1	16	2
*p*-value	0.847	
**Tumor grade**		
LG	24	3
HG	39	4
*p*-value	0.804	
**Tumor recurrence**		
Yes	10	2
No	37	3
*p*-value	0.135	
**Tumor progression**		
Yes	4	1
No	43	4
*p*-value	0.332	

**Figure 1:**
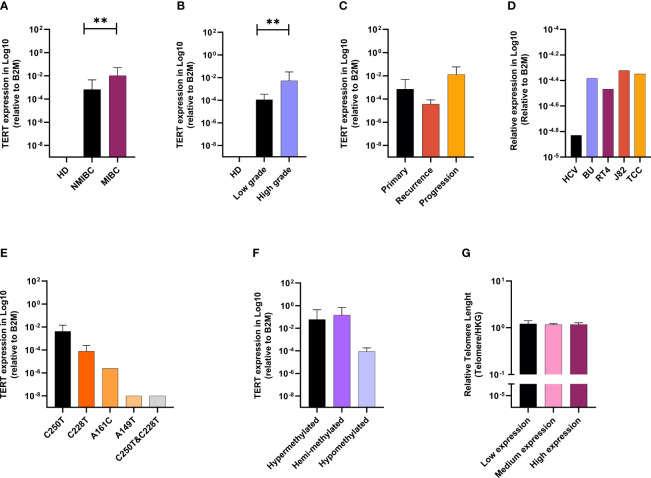
TERT is upregulated in advanced BC, linked with promotor alterations and telomere lengthening. TERT mRNA expression according to clinical stage **(A)**, grade **(B)**, and follow-up **(C)**. TERT mRAN expression in bladder cell lines **(D)**. Effect of TERT promoter mutations status **(E)** and methylation profile **(F)** on gene expression. Telomere length according to TERT gene expression **(G)**. p values < 0.01, 0.001 or 0.0001 were labelled with **, *** or ****, respectively.

We then quantified the expression of TERT in the same set of patients. The relative expression of TERT was not detected in 15 cancerous samples and in any of the healthy bladder specimens. Analysis of quantitative real-time PCR data showed that TERT was upregulated in invasive bladder stages and high tumor grade **(** **Figures 1A, B** **)**. Patients having experienced tumor recurrence upon the 4-year follow-up had lower expression than patients with tumor progression. Patients with no relapse were the ones with the highest expression level **(** **Figure 1C** **)**. In the bladder lines, the lowest expression of TERT was detected in the healthy line (HCV29), while the expression in the four cancerous cell lines was higher, but similar **(** **Figure 1D** **)**.

### C250T Mutation and Hypermethylation of TERT Promoter Drive Gene Expression in BC and Induce Telomere Lengthening

The relationship between TERT mRNA expression, TERT promoter mutation, and methylation is presented in **Figures 1E, F**. Analysis of quantitative real-time PCR data showed higher levels of TERT mRNA in cases with C228T mutation. The lowest expression level of TERT was observed in the case carrying both C228T and C250T mutations.

The analysis of TERT promoter methylation and the gene expression showed highest expression level of TERT in methylated (hypermethylated and hemimethylated) compared with unmethylated cases **(** **Figure 1F** **)**.

The relationship between telomere length (TL) and clinicopathological parameters is summarized in **Table 5**. To do that, we defined the upper limit of the overall 95% confidence interval (CI, 1.156–1.229) for 70 BC samples as a cutoff value. BC patients were categorized into two groups by using this cutoff point: short (≤1.229) and long telomeres (>1.229). In our cohort, cases with short (≤1.229) 82.86% (58/70) prevail over long telomeres (>1.229) 17.14% (12/70) **(** **Table 5** **)**. Low tumorous stage and grade were observed in both groups with long and short telomeres. Furthermore, we evaluated the correlation between telomere length and the mutational and methylation status of TERT promoter region. Results are summarized in **Table 6**. In total, 63.79% of cases carrying at least one mutation in the TERT promoter region have shorter telomeres than the 58.33% cases with no mutation. Interestingly, all cases with long telomeres and 87.93% of cases with short telomeres exhibited a methylation status of the promoter region of TERT.

**Table 5:** Distribution of telomere length according to clinicopathological parameters.

Parameters	Telomere length		
	≤1.229 (*N* = 58)	>1.229 (*N* = 12)	*p*-value
**Gender**			
Male	56	12	0.999
Female	2	0	
**Age**			
≤50	1	0	0.972
50–70	36	8	
>50	21	4	
**Smoking**			
Yes	24	4	0.784
No	34	8	
**Tumor stage**			
≤PT1	44	8	0.453
>PT1	14	4	
**Tumor grade**			
Low	23	4	0.408
High	35	8	
**Tumor recurrence**			
Yes	10	2	0.970
No	34	6	
**Tumor progression**			
Yes	5	0	0.999
No	39	8	

**Table 6:** Correlation between mutation and methylation status of TERT gene promoter and telomere length.

Telomere length	*N*	Mutational status of *hTERT* gene promoter					Methylation status of *hTERT* gene promoter				
		+		−		*p*-value	+		−		*p*-value
≤1.229	58	37	63.79%	21	36.21%	0.162	51	87.93%	7	12.07%	0.999
>1.229	12	5	41.67%	7	58.33%		12	100%	0	0%	
Total	70		42		28			63		7	

Lastly, we assessed the relationship between telomere length and TERT mRNA expression (**Figure 1G**). Telomere length was analyzed as continuous variable and TERT mRNA expression was categorized into quartiles (low expression: lower quartile, medium expression: 2nd and 3rd quartiles and high expression: upper quartile). Data analysis showed no statistically significant difference between relative telomere length and TERT mRNA expression, still with the patients’ group expressing more TERT carrying the longest telomeres.

### PD-L1 and PD-L2 Are Differentially Expressed Between NMIBC and MIBC

**Figure 2:**
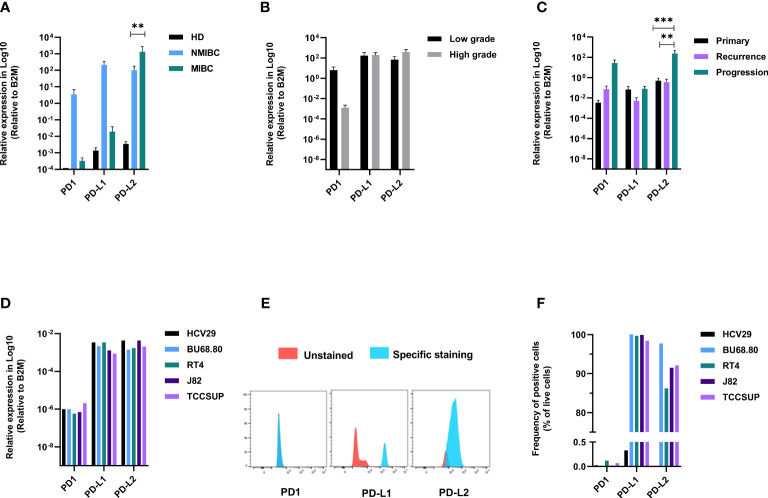
Immune checkpoints are differentially expressed between NMIBC and MIBC. PD1, PD-L1, and PD-L2 mRNA expression according to clinical stage **(A)**, grade **(B)**, and follow-up **(C)**. PD1, PD-L1, and PD-L2 mRNA expression in bladder cell lines **(D)**. Representative expression of PD1, PD-L1, and PD-L2 on bladder cell lines **(E)** and summary data of FACS analysis on the five cell lines **(F)**. p values < 0.01, 0.001 or 0.0001 were labelled with **, *** or ****, respectively.

Taking advantage of the same set of samples and to explore the potential role of telomerase on the expression of immune checkpoints, we investigated the expression of PD1, PD-L1, and PD-L2. PD1 and PD-L1 were higher in NMIBC stages (≤PT1). Conversely, PD-L2 was significantly upregulated in MIBC stages **(** **Figure 2A** **)**. Low-grade tumors showed higher expression of PD1, while the expression of PD-L1 and PD-L2 was almost similar in both low- and high-grade tumors **(** **Figure 2B** **)**. In healthy bladders, PD1 was absent or barely detectable, while PD-L1 and PD-L2 were detected at a very low level in all four specimens. A comparison between the primary, recurrent, and progression tumor groups showed that PD-L2 prevailed in the three tumor types and was upregulated in progressing tumors compared with primary and recurrent tumors **(** **Figure 2C** **)**. In the bladder cell lines, the mRNA expression of the three genes showed no significant differences **(** **Figure 2D** **)**. Instead, at protein level, no expression of PD1 was detected across the five cell lines while almost all cancerous cells, but not the healthy ones, exhibited PD-L1. PD-L2 was absent in healthy bladder cells. It scored the highest percentage of positive cells in the NMIBC line (BU68.08), while in the MIBC lines, its expression increased in parallel to the invasiveness **(** **Figures 2E, F** **)**.

### TERT/PD-L1/L2/miRNA Loop

To investigate whether miRNAs are involved in the regulation of TERT and IC, we assessed the expression of miR-100-5p and miR-135p in BC samples and bladder cell lines.

**Figure 3:**
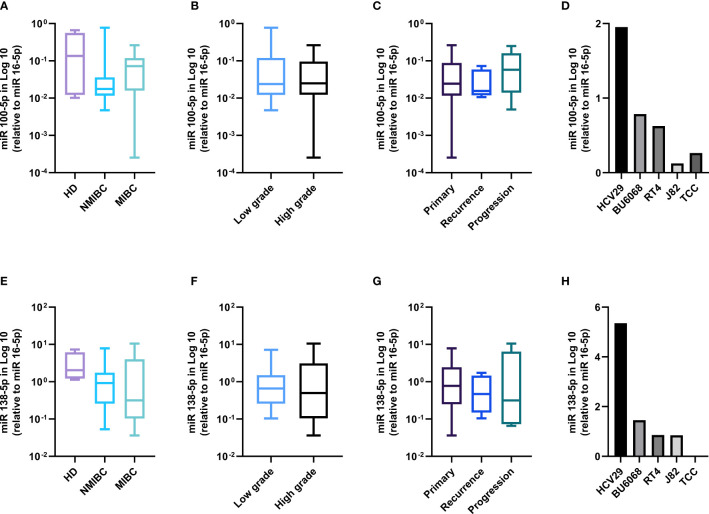
miRNAs 100-5p and 138-5p are lost in BC patients and bladder cell lines. miR100-5p expression according to clinical stage **(A)**, grade **(B)**, follow-up **(C)**, and in the bladder cell lines **(D)**. miR138-5p expression according to clinical stage **(E)**, grade **(F)**, follow-up **(G)**, and in the bladder cell lines **(H)**.

The healthy bladder tissues displayed the highest expression of both miRNAs **(** **Figures 3A, E** **)**. In the tumors, a trend was observed for higher miR-100-5p expression in the MIBC tumors as compared with the NMIBC ones **(** **Figure 3A** **)** and in the progressing tumor group as compared with primary tumors **(** **Figure 3C** **)**. The expression according to grades showed no significant difference **(** **Figure 3B** **)**. miR-138-5p was higher in the healthy tissues, decreased steadily from NMIBC to the MIBC tumors **(** **Figure 3E** **)** from low- to high-grade tumors **(** **Figure 3F** **)** and from primary to progressing tumors **(** **Figure 3G** **)**. Similarly, in the bladder cell lines, we observed that the healthy HCV29 line had the highest level of both miRNAs and that their expression level inversely correlated with the cell line tumor stages **(** **Figures 3D, H** **)**.

Next, to assess a potential effect of the miR100-5p and miR-138-5p on the expression of PD-L1, PD-L2, and TERT, we used the miRWALK database (35) to search for potential binding sites. We could confirm that miR-138 binds to TERT, PD-L1, and PD-L2 with a binding score of 1, 0.84, and 0.83, respectively. Strong validation assays (reporter assays and Western blot) performed to confirm the binding of the miR-138-5p on TERT on the 3′untranstated region and PD-L1 on the coding sequence are presented on miRTarBase (miRTarBase2020) under the respective IDs: MIRT003991 and MIRT732233. Binding data for the miR-100-5p in the same databases and in Mirabel database is still lacking.

**Figure 4:**
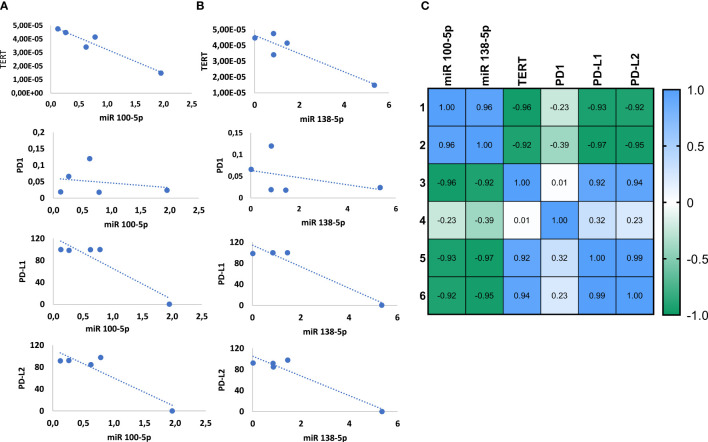
miRNAs 100-5p and 138-5p regulate the expression of TERT and PD1-ligands. Correlation of TERT mRNA level and PD1, PD-L1 and PD-L2 protein levels with miR100-5p expression **(A)** and miR138-5p expression **(B)**. Summary of Pearson correlation between the miRNAs and target genes **(C)**.

Next, we measured the Pearson’s correlation between the expression of the miRNAs and the targets in the healthy bladder and the cancerous lines and generated linearity curves **(** **Figures 4A, B** **)**. In line with the binding predictions presented above, we observed that the expression of both miRNAs inversely correlated with TERT mRNA expression and PD-L1 and PD-L2 protein expression, with an R score of −0.92, −0.93, and −0.92, respectively (*p* = 0.005, 0.012, and 0.014) for miR-100-5p and an R score of −0.92, −0.97, and −0.95 for miR-138-5p (*p* = 0.013, 0.004, and 0.006). Instead, no correlation was observed between the miRNAs and PD1 **(** **Figure 4C** **)**.

Lastly, we found that TERT expression positively correlated with PD-L1 and PD-L2. Pearson’s R score equals 0.92 for TERT and PD-L1 and 0.94 for TERT and PD-L2 arguing for a novel regulatory loop involving miRNAs, TERT, PD-L1, and PD-L2.

## Discussion

Fine tuning of gene expression, including epigenetic regulation, is key in the process of malignant transformation and for response to treatment. Here, by profiling a large cohort of BC patients, we report a novel layer of gene regulation in BC involving the loss of miR138-5p and 100-5p linked to TERT and immune checkpoint upregulation.

Several studies focused on the promoter region of TERT as a clinical biomarker, to define the factors influencing telomerase re-expression (36–38). In our prospective study, we tackled different aspect of TERT that might influence the TERT-induced carcinogenesis. We found that 60% of BC patients harbored TERT promoter mutations. These mutations were observed in the high-risk group including noninvasive tumor stage and high tumor grade and led to the highest expression of the gene at the mRNA level. Conflicting data have been reported on this aspect. Some studies reported that TERT promoter mutations in BC were significantly associated with higher tumor grades and stages (39), while others showed that TERT promoter mutations can be found in all BC stages and grades (40, 41).

The methylation of THOR was detected in 90% of cases mainly of advanced tumor stage and high grade with hypermethylated patients displaying the highest TERT mRNA levels. This suggests that the methylation profile might drive the increase of TERT expression.

TERT expression and telomerase activation play a crucial role in cancer induction and progression (42). The higher levels of TERT mRNA expression in NMIBC and in high tumor grades suggest that reactivation of telomerase may differ between superficial and invasive, between low and high grade, and between low- and high-risk BC. This pattern can be used for better patients’ stratification as it highlights tumor aggressivity and the high probability of recurrence or progression in the intermediate- to high-risk group. Furthermore, telomere length variation is strongly involved in the process of tumorigenesis (42). Telomere lengthening is associated with an increased risk of various types of cancer (43). In our investigation, cases with short telomeres prevail. This is in contrast with a study showing that patients with MIBC of any grade had shorter telomere than patients with NMIBC, of any grade (44). Therefore, we assessed the effect of TERT expression on the maintenance of the telomere length and found that patients with the highest TERT expression had the longest telomeres irrespective to the stage or grade.

Furthermore, by investigating the combined effect of TERT promoter mutation and methylation status on telomerase activation and telomere length, we report distinct telomere length categories. It has been shown that TERT promoter mutations do not prevent telomere attrition but contribute to tumorigenesis by enhancing immortalization and genomic instability in two phases. In an initial phase, TERT promoter mutations do not prevent bulk telomere shortening but extend cell lifespan by healing the shortest telomeres. In the second phase, the critically short telomeres lead to genome instability and telomerase is further upregulated to sustain cell proliferation (45).

TERT expression is upregulated in 85%–90% of tumors *via* multiple molecular mechanisms including somatic mutations, TERT structural variants, and epigenetic modifications through TERT promoter methylation (46). In our results, higher levels of TERT mRNA expression were found in cases with hypermethylation of THOR and C228T mutation in TERT promoter suggesting that TERT promoter mutations are necessary but not sufficient to maintain telomerase upregulation. In BC, TERT promoter mutation is an early event while THOR hypermethylation is associated with disease progression and increased TERT expression. This pattern has been observed in our patients who experienced tumor recurrence and progression, paralleling results obtained in other cancer types (e.g., prostate, brain tumors) (46–48).

Furthermore, we showed that the expression of PD1 and PD-L1 decreases in MIBC concomitant with PD-L2 increase. PD-L2 binds to PD1 with ∼3-fold stronger affinity compared with PD-L1 (49) and can be expressed in the absence of PD-L1 in some tumor types (50), suggesting a key immunosuppressive role of PD-L2 in the absence of PD-L1. Thus, PD-L2 expression might represent an important biomarker for better BC patients’ stratification and therapy. In an attempt to improve mono-immunotherapies, it was reported that the use of telomerase-specific oncolytic virotherapy can enhance the efficacy of PD1 blockade in osteosarcoma (51). Another study showed that TERT can increase tumor immunogenicity and affect the response to ICI therapy (13). An explanation for these clinical observations stems from our correlations between TERT and PD-L expression in BC patients. In the NMIBC, we observed that patients with high TERT levels also express the highest level of PD-L1, while in the MIBC stages, TERT was paired with PD-L2 expression. PD-L2 was also upregulated in the progression group. Moreover, in the BC cell lines, we detected a positive correlation between TERT and PD-L2 across all tumor stages, suggesting a TERT-PD-ligand regulatory loop that might confer to tumor cells expressing high levels of PD-L1/L2 a longer lifespan through telomerase reactivation.

In that context, miRNAs are key in the regulation of gene expression and as noninvasive sensitive and specific plasma biomarkers for BC diagnosis and prognosis (52). Low miR-100 expression has been shown to inversely correlate with FGFR3 expression, longer PFS, and cancer-specific overall survival in BC. High levels of miR-138-5p were associated with positive cyclin D3 protein expression in BC patients (22, 53). Thus, we measured the level of these two miRNAs in our cohort, in relationship to TERT and PD1 ligands. The observed reduction of these miRs along with tumor progression in the high-risk patients, the linearity study showing an inverse correlation with the expression of PD-Ls and the presence of binding sites on the target genes suggest that the miR100-5p and miR-138-5p might play a central role in the regulation of the expression of both TERT and immune checkpoint ligands. On a translational perspective, recent advances in BC-targeted therapy using miRNAs have demonstrated the usefulness of intravesical instillation of miR-143 and miR-145 in inhibiting tumor growth and proto-oncogene silencing (54, 55). Therefore, we speculate on the potential clinical benefit of intravesical administering miRNAs to improve the effect of immunotherapy, by concomitantly limiting systemic toxicity. Overexpression of these miRNAs in the tumor microenvironment might enhance immunogenicity and improve the outcome of ICI therapies, not only in advanced cases but also in cisplatin ineligible, in primary or recurrent NMIBC where high level of TERT and PD-L1 can be detected.

We are aware that the limitations of this study lie in the relatively small sample size of our cohort, which may have limited the statistical power of our analysis, and in the impossibility to correlate our finding with the response to ICIs in the same set of patients as none of the participants included in our cohort could benefit from these immunotherapies. Future studies should aim at extending our findings by evaluating the same genes and miRs in cohorts of ICI-treated BC patients, to refine treatment options and dissect resistance mechanisms in BC.

Overall, our findings directly in BC patients emphasize the use of TERT, PD-L1, PD-L2, miR-138-5p, and miR-100-5p as diagnosis and prognosis biomarkers for patients’ stratification and follow-up. Thus, in BC patients, the combination of miRNAs targeting ICIs and TERT by direct instillation in the bladder can provide a promising therapeutic strategy for an *in situ* treatment.

## Data Availability Statement

The original contributions presented in the study are included in the article/supplementary material. Further inquiries can be directed to the corresponding author.

## Ethics Statement

This study protocol was reviewed and approved by the Ethics Committee for Biomedical Research from the Faculty of Medicine and Pharmacy of Rabat-Morocco (N82/19) and University Center of Legal Medicine Lausanne-Geneva, University of Lausanne, Lausanne-Switzerland (VD431/13). The patients/participants provided their written informed consent to participate in this study.

## Author Contributions

HE designed the study, performed the experiments, analyzed the data, and wrote the paper. MA analyzed the data and contributed to the paper writing. CA, IH, and IC performed biostatistical analysis. MT, AA, MO, and KM provided patient samples and follow-up. AA, MB, and LB provided intellectual contributions and revised the manuscript. MA provided intellectual contribution and critically revised the manuscript. CJ and MA provided intellectual contribution, supervised all the experiments, critically revised the manuscript, and gave final approval to the publication.

## Funding

This work was supported by grants from the Swiss National Science Foundation (PRIMA PR00P3_179727) to CJ.

## Conflict of Interest

The authors declare that the research was conducted in the absence of any commercial or financial relationships that could be construed as a potential conflict of interest.

## Publisher’s Note

All claims expressed in this article are solely those of the authors and do not necessarily represent those of their affiliated organizations, or those of the publisher, the editors and the reviewers. Any product that may be evaluated in this article, or claim that may be made by its manufacturer, is not guaranteed or endorsed by the publisher.
